# suPAR cut-offs for stratification of low, medium, and high-risk acute medical patients in the emergency department

**DOI:** 10.1186/s12873-021-00544-x

**Published:** 2021-11-29

**Authors:** Seppälä Santeri, Andersen Andreas Peter, Nyyssönen Kristiina, Eugen-Olsen Jesper, Hyppölä Harri

**Affiliations:** 1 South Savo Social- and Healthcare District, Mikkeli Central Hospital, Mikkeli, Finland; 2 Department of Clinical Research, Copenhagen University Hospital Amager and Hvidovre, Hvidovre, Denmark; 3 Eastern Finland Laboratory Centre Joint Authority Enterprise (ISLAB), South Savo District Laboratory, Mikkeli, Finland

**Keywords:** suPAR, Laboratory marker, Emergency department, Mortality

## Abstract

**Background:**

Soluble urokinase plasminogen activator receptor (suPAR) levels have previously been associated with readmission and mortality in acute medical patients in the ED. However, no specific cut-offs for suPAR have been tested in this population.

**Methods:**

Prospective observational study of consecutively included acute medical patients. Follow-up of mortality and readmission was carried out for 30- and 90 days stratified into baseline suPAR < 4, 4–6 and > 6 ng/ml. suPAR levels were measured using suPARnostic® Turbilatex assay on a Cobas c501 (Roche Diagnostics Ltd) analyser.

**Results:**

A total of 1747 acute medical patients in the ED were included. Median age was 70 (IQR: 57–79) and 51.4% were men. Adjusted linear regression analysis showed that suPAR, independently of age, sex and C-reactive protein levels, predicted 30- and 90-day mortality (Odds ratio for doubling in suPAR 1.96 (95% confidence intervals: 1.42–2.70) Among patients with suPAR below 4 ng/ml (*N* = 804, 46.0%), 8 (1.0%) died within 90-day follow-up, resulting in a negative predictive value of 99.0% and a sensitivity of 94.6%. Altogether 514 (29.4%) patients had suPAR of 4–6 ng/ml, of whom 43 (8.4%) died during 90-day follow-up. Among patients with suPAR above 6 ng/ml (*N* = 429, 24.6%), 87 patients (20.3%) died within 90-day follow-up, resulting in a positive predictive value of 20.1% and a specificity of 78.7%.

**Conclusions:**

suPAR cut-offs of below 4, between 4 and 6 and above 6 ng/ml can identify acute medical patients who have low, medium or high risk of 30- and 90-day mortality. The turbidimetric assay provides suPAR results within 30 min that may aid in the decision of discharge or admission of acute medical patients.

## Background

Clinical and laboratory markers of diagnosis and prognosis are needed to safely and quickly distinguish between high-risk acute medical patients who will require admission to hospitals and low-risk patients who can be discharged to recover in another institution or in their private home.

[1]. Often, risk scores such as the Early Warning Score which is developed for 24-h in-hospital mortality is used for triage. However, a limitation of currently used risk scores that are based on clinical signs is the inability to identify patients with largely unaffected clinical signs who still have a high risk of 30- or 90-day mortality. Thus, there is an opportunity for improving triage by including knowledge of patient prognosis. This could lead to more low risk patients being early and safely discharged, thereby reducing patient crowding, and to avoidance of premature discharge of high-risk patients [1, 2].

There are several reasons why soluble urokinase plasminogen activator receptor (suPAR) is a suitable attention biomarker in unselected acute medical patients: First, suPAR is a non-specific marker elevated by diseases in general and by the severity of disease. suPAR has prognostic value in patients without any comorbidity, as well as in patients with comorbidity, such as Type 1 Diabetes [3], and Type 2 Diabetes [4], cardiovascular disease [5] and chronic obstructive pulmonary disease [6]. Secondly, suPAR is a stable marker and the measurement is unaffected by diurnal changes [7], and thirdly, suPAR has recently become easy to measure using automated turbidimetric analysis providing fast answers on overall patient prognosis [8].

The clear advantage regarding suPAR results is when the patient has a low suPAR, indicating that the patient has a well-functioning overall immune response, a low risk of presence of severe disease and a low risk of readmission and mortality [9]. In agreement with this, a randomised controlled study of making suPAR available to clinicians or not with more than 16 thousand acute medical patients showed significant more patients were safely discharged in the suPAR arm compared to the control arm [10]. Early discharge of acute medical patients can free up bed capacity and lower the pressure on hospital staff and this may be more important than ever, considering the ongoing SARS-CoV-2 pandemic. Also in COVID-19, suPAR has been shown to be a strong marker of disease development, with high risk of respiratory failure in patients with suPAR above 6 ng/ml [11].

Despite intensive research in suPAR, clear guidelines on suPAR cut-offs are not available.

At Copenhagen University Hospital Hvidovre, Denmark, suPAR has been measured in acutely admitted medical patients at the Emergency Department (ED) since 2013 [12]. In studies conducted before the COVID-19 pandemic, among unselected acute medical patients, it was found that suPAR < 3 ng/ml is associated with low risk of readmission and mortality (approximately half of the admitted acute care medical patients), 3–6 ng/ml as medium risk, and > 6 ng/ml as high risk requiring clinical attention [12]. As there is a linear correlation between suPAR and outcomes, the lower the suPAR level, the less the risk of a negative patient outcome. However, if the cut-off is set to low, the number of patients will be too few to have clinical impact in triage of ED patients. Many studies have been carried out on quartiles or log2-transformed suPAR values, but the use of specific cut-offs would allow for comparison and possible reproduction of findings in independent studies. A position paper from the Hellenic Sepsis study group suggested suPAR below 4 ng/ml for discharge and above 6 ng/ml for further examination [13]. These cut-offs were recently tested in patients with symptoms of COVID-19, showing low risk of adverse outcomes in patients with suPAR below 4 ng/ml and high risk in patients with suPAR above 6 ng/ml [14]. Furthermore, the cut-off of 6 ng/ml was used as inclusion criteria for the suPAR-guided anakinra trial (SAVE MORE) in COVID-19 [15].

We aimed to investigate whether suPAR and the specific cut-offs of suPAR can be used for risk stratification of Finnish patients seeking care at the Emergency Department. We aimed to determine the negative predictive value of a low suPAR (≤ 4 ng/ml) for readmission and mortality, and secondly, to evaluate whether elevated suPAR levels (≥ 6 ng/ml) is associated with high risk of a negative outcome.

## Patients and methods

A prospective cohort study of consecutively acute medical patients seeking care at ED at Mikkeli Hospital in Finland from 4th of March 2020 to 11th of May 2020 and had blood samples taken for routine biomarkers (*n* = 1747). At admission, each patient who had a standard panel of blood tests analysed had suPAR added to these standard blood tests. Only patients who had blood samples taken from them were included in the study. So, the study did not include patients with only covid test taken or patients with minor injuries, minor medical problems, nurse visits, children, phone calls or mental health problems.

Mikkeli Central Hospital is the nearest hospital for approximately 98,000 people and therefore sees all kinds of emergency patients in the ED. Annually there are 50,000–55,000 patient visits (54,031 patient visits in 2019) and admission rate is usually approximately 20–23% (10,881 patients admitted in 2019).

**Fig. 1:**
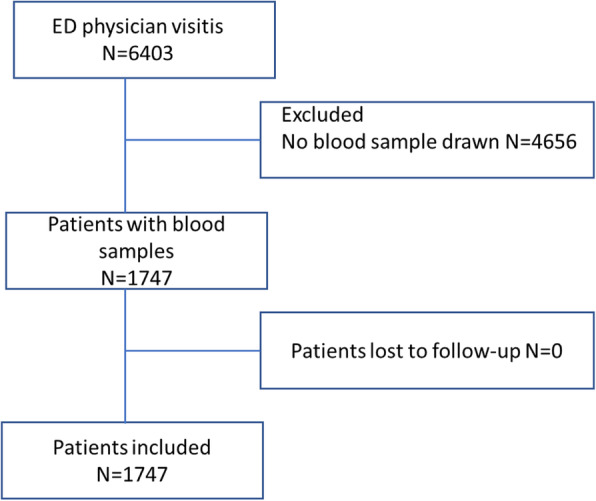
Flow chart of included patients

During the study time Mikkeli Central Hospital had 6403 patient visits. Detailed description of patient visits is shown in Fig. 1. Patients, who only had COVID-19 swaps taken were categorized as internal medicine patients, but were not part of the study population.

### Data

Data was stored individually in each patient health record and was transferred from the hospital electronic health record system (Effica) to Excel.

### Biomarker measurements

Plasma suPAR levels were analysed as part of the standard admission blood samples at the Eastern Finland laboratory ISLAB with the using suPARnostic® Turbilatex assay (ViroGates A/S, Birkerød, Denmark) on a Cobas c501 clinical chemistry analyser (Roche Diagnostics Ltd) according to the reagent manufacturer’s instructions. Plasma C-reactive protein levels (CRP) and plasma creatinine were also measured with the Cobas c501 analyser from the same blood sampling tube as plasma suPAR. Blood lymphocyte count was measured by Advia® 2010 Hematology analyser (Siemens Healthcare Diagnostics Inc., NY, USA).

### Endpoints

The primary endpoint was mortality, and analysis was carried out for 7-, 30- and 90-day mortality, respectively. Secondary endpoint was readmission, and analysis was carried out for 30- and 90-day, respectively. Furthermore, patients that had a prior admission up to 30-days before baseline were analysed for whether they had elevated suPAR at baseline compared to patients without an admission 30-days prior to baseline.

Deaths were recorded from local EMR which is linked to national log of death certificates and therefore includes all deaths recorded in Finland. All patient data (including deaths and readmissions) is also linked to local EMR from Finnish national health care record (KANTA) and therefore deaths and readmissions are checked from all Finnish healthcare providers.

### Statistical analysis

The *p*-values given are tests of normality, with age and suPAR using a Shapiro-Wilk test of normality. The rest are binomial factors and are tested with a Pearson chi-squared test.

ROC curves (receiver operating characteristic curves) are created in R, using the ROSE package1. The true positives and false positives are compared, using CRP, suPAR, age, on 90-day mortality as outcome. The area under the curve (AUC) is given with 95% Confidence Intervals (CI) calculated by the DeLong test.

Two linear regression models were created using 90-day mortality as the binomial outcome. The first included linear suPAR (log2) adjusted for CRP (log10), sex, and age. The second included categorised suPAR using the cut-offs < 4, 4–6 and > 6 ng/ml adjusted for age, sex and CRP (log 10). The Odds ratios are shown with 95% confidence intervals.

Significance was set at the 5% level. All data was processed and analysed using R (R Core Team (2020). R: A language and environment for statistical computing. R Foundation for Statistical Computing, Vienna, Austria. (URL https://www.R-project.org/).

## Results

A total of 1747 acute medical patients were included. Median age was 70 (IQR: 57–79) and 51.4% were men. Table 1 shows baseline characteristics for all patients and for patients stratified by baseline suPAR < 4, ≥4 - ≤6 and suPAR > 6 ng/ml. Almost half of the patients (48.3%) had a suPAR below 4 ng/ml. suPAR increases with age whereas sex did not change significantly across the groups (Table 1). Patients that presented with comorbidities (Diabetes 1 or 2 (DM), cardiovascular disease (CVD), neurological disease (NEU) or pulmonary disease (PULM) had generally elevated suPAR levels (Table 1).

**Table 1:** Baseline characteristics

	All	suPAR < 4	suPAR 4–6	suPAR > 6	***P*** value
N (%)	1747	804 (46.0)	514 (29.4)	429 (24.6)	
Age median (IQR)	70 (57–79)	62 (44–73)	74 (65–83)	76 (67–85)	< 0.001
Sex (=male %)	897 (51.4)	436 (55.2)	250 (48.6)	211 (49.2)	0.08
suPAR ng/ml (IQR)	4.1 (3.3–6.0)	3.2 (2.9–3.6)	4.7 (4.3–5.3)	8.5 (7.1–11.3)	< 0.001
CRP ug/ml (IQR)	3 (3–17)	3 (3–4)	4 (3–18)	20 (5–72)	< 0.001
Lymphocyte count 10^3^/ul (IQR)	7.5 (6.0–9.8)	7.3 (5.9–8.8)	7.7 (5.9–9.8)	8.55 (6.3–12.1)	< 0.001
Creatinine (IQR) nmol/ml	77 (64–96)	70 (61–83)	79.5 (65–97)	101 (74–139)	< 0.001
**Comorbidities**					
Diabetes N (%)	365 (20.9)	109 (13.6)	121 (23.5)	135 (31.5)	< 0.001
Cardiovascular disease N (%)	1151 (65.9)	416 (51.7)	387 (75.3)	348 (81.1)	< 0.001
Neurological disorder N (%)	516 (29.5)	209 (26.0)	156 (30.4)	151 (35.2)	0.003
Pulmonary disease N (%)	388 (22.2)	155 (19.3)	126 (24.5)	107 (24.9)	0.024

Age, suPAR (soluble urokinase plasminogen activator receptor), CRP (C-reactive protein), Lymphocyte count and Creatinine were tested with Shapiro-Wilks test. Sex, Diabetes, Cardiovascular disease, Neurological disease and Pulmonary disease were tested with Pearson Chi-squared test. IQR: Interquartile range

### Readmission and suPAR

In all 379 (21.7%) of the patients had been admitted to the hospital within 30-days prior to the study inclusion. Patients that had a prior admission had higher suPAR levels at baseline (29.6% among patients with suPAR above 6 ng/ml versus 16.3% of patients with suPAR below 4 ng/ml, *p* < 0.001, Table 2). In contrast to this, there was no significant difference in 30-day readmission following baseline suPAR measurement (23.9% among patients with suPAR above 6 ng/ml versus 19.0% of patients with suPAR below 4 ng/ml, *p* = 0.14) (Table 2).

**Table 2:** Outcomes and outcomes in relation to suPAR cut-offs

	All	suPAR < 4 ng/ml	suPAR 4–6	suPAR > 6 ng/ml	P value
N	1747	804	514	429	
Discharge < 24 H N (%)	785 (44.9)	462 (57.5)	215 (41.8)	108 (25.2)	< 0.001
30 Day pre-admitted N (%)	379 (21.7)	131 (16.3)	121 (23.5)	127 (29.6)	< 0.001
Readmission 30 Days N (%)	368 (21.1)	153 (19.0)	114 (22.2)	101 (23.5)	0.14
Mortality 7 Days N (%)	34 (1.95)	3 (0.37)	9 (1.75)	22 (5.13)	< 0.001
Mortality 30 Days N (%)	81 (4.64)	6 (0.75)	25 (4.9)	50 (11.7)	< 0.001
Mortality 90 Days N (%)	138 (7.90)	8 (1.0)	43 (8.4)	87 (20.3)	< 0.001

All P-values were calculated using the Pearson Chi-squared test. 30 Day preadmitted refers to number of patients that had an admission to hospital within 30-days prior to the baseline suPAR measurement

### Association with suPAR and 30 and 90-day mortality

During 30-day follow-up, 81 (4.6%) patients died and this number increased to 138 (7.9%) after 90 days. Patients with suPAR below 4 ng/ml had lower risk of mortality, both with regard to 30- and 90-day mortality (both *p* < 0.001, Table 2). With regard to 90-day mortality, we observed a 20-fold higher mortality in patients with suPAR above 6 ng/ml (87 died out 429, 20.3%), compared to below 4 ng/ml (8 died out of 804 patients, 1.0%) (Table 2).

### Prediction of 90-day mortality using ROC AUC analysis

**Fig. 2:**
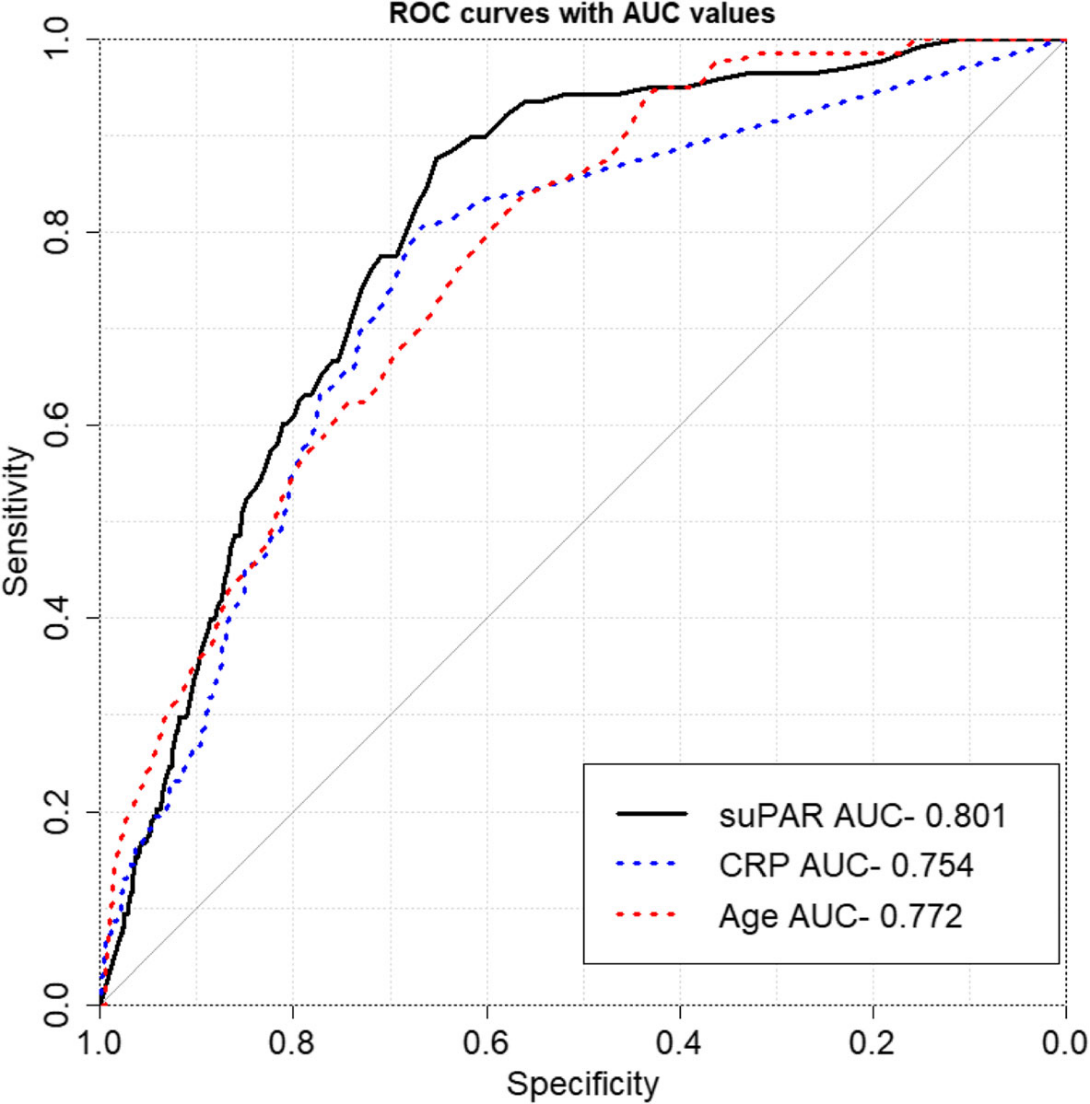
Receiver Operating Curves (ROC) of Age (Red stippled line), CRP (Blue stippled line) and suPAR (Black line) with regard to 90-day mortality. The Area Under the Curve (AUC, 95%CI) for age is 0.77 (0.74–0.81), CRP is 0.75 (0.71–0.79) and suPAR is 0.80 (0.77–0.83). AUC: Area Under the Curve. CRP: C-reactive protein. suPAR: Soluble urokinase plasminogen activator receptor

To compare the predictive values of age, CRP and suPAR for 90-day mortality, we used ROC analysis and calculated the Area under the Curves (AUC). As shown in Fig. 2, age, CRP and suPAR were all predictive of 90-day mortality, with AUC’s (95%CI) of 0.77 (0.74–0.81), 0.75 (0.71–0.79) and 0.80 (0.77–0.83), respectively.

### Adjusted regression analysis

To determine whether the association between suPAR and mortality was independent of age, sex, and CRP levels, multivariate linear regression analysis was carried out including log2 suPAR (per 100% increase (doubling) in suPAR). For every doubling in suPAR, the Odds ratio for 90-day mortality increased with 1.96 (95% confidence intervals: 1.42–2.70) showing that suPAR independent of age sex and CRP was associated with 90-day mortality. Univariate and mutually adjusted odds ratios for each of the variables are shown in Table 3.

**Table 3:** Logistic regression analyses for 90-day mortality

Characteristic	Crude OR	95% Conf. Int.	Adjusted OR	95% Conf. Int.	Sign.
Age	1.09	(1.07–1.11)	1.08	(1.06–1.10)	< 0.001
Sex	0.80	(0.57–1.14)	0.73	(0.49–1.07)	0.109
CRP (log10)	3.99	(3.05–5.22)	2.88	(2.07–4.04)	< 0.001
suPAR (log2)	3.91	(3.08–4.99)	1.96	(1.42–2.70)	< 0.001

Crude OR is univariate logistic analysis for 90-day mortality. Adjusted is variable of interest holding others constant. Significance p-value is for adjusted odds ratio

Compared to having suPAR below 4 ng/ml, patients with suPAR above 6 ng/ml had a sex- and age-adjusted Odds ratio of 13.2 (95%CI: 6.6–30.3). Patients with suPAR equal to or between 4 and 6 ng/ml had an increased Odds ratio of 2.4 (95%CI: 1.6–3.7) compared to patients with suPAR below 4 ng/ml.

### Sensitivity, specificity, NPV and PPV for suPAR cut-offs and 30- and 90-day mortality

Of the 804 patients with suPAR below 4 ng/ml, 6 died (0.7%) within 30-days of admission resulting in a negative predictive value of 99.3%. Among patients with suPAR above 6 ng/ml (*N* = 429), 50 patients died within 30 days (11.7%), corresponding to a positive predictive value of 11.6% and a specificity of 77.4%. With regard to 90-day mortality, 8 out of 804 (1.0%) with suPAR below 4 ng/ml died resulting in a NPV of 99.0% and a sensitivity of 94.6%. In patients with suPAR above 6 ng/ml, a PPV of 20.1% and a specificity of 78.7% was observed. Tables 4 and 5 shows sensitivity, specificity and NPV and PPV for 30- and 90-day mortality at the suPAR cut-off of 4 ng/ml and suPAR cut-off at 6 ng/ml, respectively.

**Table 4:** NPV, PPV, sensitivity and specificity at cut-off 4 ng/ml. NPV: Negative predictive value; PPV: Positive Predictive value

	All	suPAR < 4 ng/ml	suPAR = > 4 ng/ml	NPV %	PPV %	Sensitivity %	Specificity %
N	1747	804	943				
Mortality 30 Days N (%)	81 (4.6)	6 (0.75)	75 (8.0)	99.3	7.95	92.6	47.9
Mortality 90 Days N (%)	138 (7.9)	8 (1.0)	130 (13.8)	99.0	13.8	94.2	49.5

NPV: Negative predictive value; PPV: Positive Predictive value

**Table 5:** NPV, PPV, sensitivity and specificity at cut-off 6 ng/ml. NPV: Negative predictive value; PPV:Positive Predictive value

	All	suPAR < = 6 ng/ml	suPAR > 6 ng/ml	NPV %	PPV %	Sensitivity %	Specificity %
N	1747	1318	429				
Mortality 30 Days N (%)	81 (4.6)	31 (2.4)	50 (11.7)	97.6	11.7	61,7	77,3
Mortality 90 Days N (%)	138 (7.9)	51 (3.9)	87 (20.3)	96.3	20.3	63.0	78,7

## Discussion

In the current study, we measured suPAR using a new turbidimetric assay allowing for suPAR values along with other biomarkers. We tested previously suggested cut-offs of suPAR that may indicate low, medium and high risk of 30- and 90-day follow-up [13]. Among the 1747 acute medical patients included, almost half had a suPAR level below 4 ng/ml, and the 30- and 90-day risk of mortality in these patients were below 1%. In contrast, patients with suPAR above 6 ng/ml (1 in 4 patients) had a high 90-day mortality of 20%. These data suggest that a suPAR level below 4 ng/ml seems useful as a potential discharge biomarker may be part of a decision to discharge the patient.

suPAR is a nonspecific biomarker, reflecting the level of chronic inflammation in the patient. suPAR is elevated by disease in general, as also reflected in this study, where we observed increased suPAR levels in patients with comorbidities.

It has previously shown that low suPAR is associated with low risk of adverse outcomes for acute medical patients, such as acute kidney injury [16, 17], acute surgery [18], and overall mortality [12]. These studies were mainly retrospective biobanked studies using ELISA platform for measurement of suPAR in batches, which demands manual work and has high processing time. In the current study, using a new fully automated turbidimetric analysis of suPAR, a NPV of 99,3% for 30-day mortality was observed in patients with suPAR below 4 ng/ml, indicating that a low suPAR may add in the decision to discharge the patient. It should be emphasized that only patients with more severe symptoms are referred to the ED for blood samples and examination, and that no patients should be discharged without through clinical examination. Nevertheless, our present results suggest that suPAR, measured with a short turnaround time, may be useful tool for risk stratification of patients.

We observed that patients that had been admitted to hospital within 30 days prior to the study inclusion had higher levels of suPAR compared to patients that had no prior 30-day admissions. In contrast to this, we found no significant association between baseline suPAR and readmissions following the index measurement. There may be several interesting explanations for this observation. First, those with prior admissions had to survive until the index admission, whereas patients following the index admission may die before readmission (mortality competes with readmission). Another possible explanation is that those who had a previous admission had become more severely ill and were therefore readmitted and this was reflected in the elevated index admission suPAR level.

Triage in the ED has become even more important during the COVID-19 epidemic, but only seven patients had a SARS CoV-2 positive test in the current study. However, suPAR has emerged as an important marker for development of respiratory failure and acute kidney injury in COVID-19 [11, 19]. Similar to our study, a suPAR cut-off of above 6 ng/ml has been suggest as cut-off for identification of COVID-19 patients at high risk for negative outcome and a suPAR level above 6 ng/ml was used as inclusion criteria for anti-inflammatory treatment with an IL-1 receptor blocker, Anakinra [20].

The emergency departments are often overcrowded and we need new methods for the risk stratification of the patients. For clinicians it is important to find new ways to safely discharge patients. suPAR is an interesting, novel biomarker, which could be used more actively in the emergency departments in patient stratification. Promising results have been reported especially with patients with low suPAR and also our study shows that low suPAR level can be a helpful clinical tool in making discharge decisions. Discharge of patients with low suPAR plasma levels seems to be safe, if other clinical judgement is also supporting discharge. Further studies and experiences are still needed to clarify the role of suPAR at the ED. Especially important is to know, how does suPAR work in different age groups.

There were limitations of the study. First, we did not record smoking habits of the patients, and smoking is a known elevator of suPAR with approximately 1 ng/ml raise compared to non-smokers [21]. Secondly, suPAR results were available to the staff in the ED along with the other biochemistry results, but it is unknown if any clinical decisions were made based on suPAR. Thirdly, this is a single centre study and most patients included were of Caucasian ethnicity due to the homogeneous Finnish population.

## Conclusions

This study shows that suPAR, measured using a fully automated turbidimetric assay, provides prognostic patient value. In our study of acute medical patients, we find that patients with a suPAR level below 4 ng/ml have low risk of 30- and 90-day mortality (less than 1%), whereas 90-day mortality reached above 20% in patients with suPAR above 6 ng/ml. This high negative predictive value in patients with suPAR level below 4 ng/ml can aid in the decision to discharge of patients, in combination with other clinical findings. We have furthermore suggested cut-offs for the use of suPAR and risk of mortality in acute medical patients. The proposal of cut-offs of suPAR for acute medical patients seeking care in the Emergency Department allows other to test the NPV’s, PPV’s sensitivity and specificity at the respective cut-offs.

In conclusion, suPAR can be measured alongside other markers using a routine clinical chemistry analyser and the result provides valuable information on 30- and 90-day risk of mortality in acute medical patients. Our suggestion of cut-offs allows for suPAR guideline development following replication in independent studies.

## Data Availability

The datasets generated during the current study are not publicly available due to National data protection agency rules but are available from the corresponding author on reasonable request and approval by the National Data Agency.

## Abbreviations

suPAR: soluble urokinase plasminogen activator receptor
ED: Emergency department
CRP: C-reactive protein
ROC curves: receiver operating characteristic curves
DM: Diabetes
CVD: cardiovascular disease
NEU: Neurological disease
PULM: Pulmonary disease
AUC: Area under the Curve
PPV: Positive predictive value
NPV: Negative predictive value
